# The urologist's role in the fight of COVID-19 pandemic: mandatory mindset shift on the frontline

**DOI:** 10.1590/S1677-5538.IBJU.2020.0316

**Published:** 2020-07-31

**Authors:** Alexandre Iscaife, Giovanni S. Marchini, Victor Srougi, Fabio C. M. Torricelli, Alexandre Danilovic, Fabio C. Vicentini, Marcos Machado, Marcelo Hisano, Bruno C. Tiseo, Júlio C. Bissoli, Marcello Cocuzza, Jorge Hallak, Miguel Srougi, William C. Nahas

**Affiliations:** * Faculdade de Medicina da Universidade de São Paulo Hospital das Clínicas Divisão de Urologia São Paulo SP Brasil Divisão de Urologia, Hospital das Clínicas, Faculdade de Medicina da Universidade de São Paulo, SP, Brasil

*To the editor,*

Early in January 2020, we were living our regular lives. We are urologists at the “Hospital das Clínicas”, University of Sao Paulo Medical School Clinical Hospital (HC-FMUSP), a 900-bed tertiary care facility inside an even wider and stronger health complex of 2.300 beds. Our Institution is responsible for about 350 highly complex monthly urological surgeries comprising all subspecialties in the field. We are inserted in the SUS (Sistema Único de Saúde), a unified health system with universal coverage created in 1990 (1). By the end of January 2020, we were attending crowded outpatient ambulatories, performing our usual surgeries, participating in the teaching of undergraduate students, teaching general surgery and urology residents, presenting scientific research in national and international scientific meetings.

When the press released the first confirmed case of corona virus disease (COVID-19) in late December, 2019 by the previously named “Wuhan virus” (Hubei province in China), later renamed as SARSCoV-2 (2), not even the most pessimistic of us could imagine the impact it would have on our lives. Not even when it was spotted that the virus spread was hidden from the World and that doctors who tried to disseminate the information out died of the disease. At worst, if the virus crossed oceans and continents to our country, the routine of clinicians working in emergency rooms and intensive care units could be affected. Important and influential individuals of our national medical and political society came to the public warning that there was nothing to fear or to worry about. They told, we believed.

Those who lived to see April 2020 realized how naive we had been. Italy fell and the whole World kneeled together with the Mediterranean country. “No man is an island, entire of itself; every man is a piece of the continent, a part of the main. If a clod be washed away by the sea, Europe is the less” (3). How can the wisdom of a 17th century man be so contemporary?

Perhaps because true wisdom is beyond time. While Italy was bleeding, here in Brazil most of our surgeries started to be postponed and the outpatient clinics to be rescheduled based on international and national urological societies recommendations (4). Academic meetings were canceled indefinitely. One by one, all the familiar aspects of our lives were taken away from us. Whilst we could still get together in the cafes, we were astonished. What´s next?

Heartbreaking videos came from all over the World: first Italy, then Spain, England, and finally the United States. Great nations with robust health care systems took a hit like they have never seen before, at least not since the World War II. The context of a locally Chinese isolated crisis was rapidly dissipated into thin air. Thunders of war were quickly moving in our direction. It was real, surreal, and we did not plan for it. On March 24, the state of Sao Paulo officially announced social isolation. Cancelation of all sports events. Empty streets. Parks with closed gates. Children and parents trapped at home. We lost freedom as we knew it.

On April 7, our institution determined the reorganization of our facilities and the mandatory internal social distancing. We could no longer meet at the cafes. Our hospital, the largest in Latin America, initiated a war operation to receive patients with COVID-19 from all over the state. One building, 900 beds, and our greatest specialists of internal and intensive care medicine, pneumology, infectious diseases and anesthesiology were assigned to assist patients who were arriving by the dozens. The number of intensive care unit beds doubled and were immediately filled. All residents of all specialties were assigned to take care of these patients. Surgical and clinical specialties not related to COVID-19 were moved to other facilities within the complex. A significant reduction in all personnel daily activities to what was strictly essential, keeping an eye in the upcoming tsunami. Potential shortage of resources, tests and support equipment started to be of concern, irrespective of having the monetary funds or not, as an international hidden cold war was on the way to get vital equipment first. Chairmen from all departments and medical specialties managed to coordinate private donations from all over the country. Large companies, banks, financial institutions, and even middle-class citizens gave significant amounts of capital to support the hospital in a severe calamity like the one to come.

Whatever our thoughts were regarding the validity and effectiveness of social distancing, total quarantine was imposed. At that point, a feeling of uncertainty was in the air (5). Where should we go now? How do we continue to treat our patients? However, that specialist-based thinking that was still with us was about to change. The first mentality shift was about to take place. Inside our hospital all fit for the battle were selected to work in the coronavirus area. Irrespective of their initial status or division. There was no intermediate position anymore: you were either considered a COVID doctor, or a COVID-free doctor. There was no turning back. The Urology Division was asked to help further. In addition to providing the 40 beds of the ward, a group of urologists was assigned to participate in the direct care of patients with COVID-19 infection. We should lead a team of residents that included those from urology, under direct supervision of a clinician. We all remember the exact moment we received the call that gave us the assignment. There was not much time for us to question the reality: it was because they needed it, and because they trusted us. A given task is an accomplished mission. That is our nature as urologists. The “Hippocratic Oath” spoke louder within our souls.

After the first shock, we aimed for objectivity. Okay, how are we going to do that? A WhatsApp group was created and sharing of relevant information began. Institutional protocol for the diagnosis and treatment of COVID-19, the main publications in the area, podcasts, videos, preparation courses. Appropriate vestment? We already know how to do surgical dress up. But do we really? It is in the removal of the vestment that most of us become contaminated. Ok then, vestment removal courses: check! Intubation course with rapid sequence, indication of correct sedatives, neuromuscular blockers, no manual ventilation, mechanical ventilation, etc. Each hour that approached us of the first shift made evident how far we were from the internal medicine and advanced clinical care management. Anxiety was second to the strict sense of duty as physicians. Everyone had heard of someone without comorbidities as young as us who needed mechanical ventilation. Deadly virus, especially for the old ones. Fear grew. How would we protect our families from ourselves? Once responsible for our children, parents and significant others, now we had become a threat. Should we leave our homes? Who will look after them for us? A feeling of impotence rose. The kind of thrill we are not used to anymore.

The day before the first shift, insecurity: we had been urologists for so long, super specialists, how would it be practicing internal medicine again? Fifteen or more years ago, each one of us, for an intimate and individual reason, made a choice for the profession of faith within medicine: we would be urologists. Our main strength was this after all. And it would be as urologists that we would face this aberrant situation. Without realizing it, when we confronted the problem dispassionately, we were already being the urologists that we always were. Let the battle begin.

On the first shift, the ritual of dressing up and the look of the residents was uncomfortable: in their eyes, our same many fears as looking into a mirror of our soul. But they were younger. And we, in distress, were the reference. The only thing to do in such scenario: start working. With one relief, the colleague from the internal medicine was there. We had been fundamentally independent for so long that a peculiar former mood started to take place. We had our masters, of course, but part of the wisdom of those figures necessary for our professional autonomy was already introjected into us. We recalled the most primordial learning processes and remembered the relief that our teachers brought to the operating field when we lost the surgical plan or there was too much blood on it.

A reunion with humbleness, while still being practical: what could we offer residents and patients? We got accustomed to precision and only accept nothing less than highly successful results with fewer complications each year. Potency and continency after radical prostatectomies; microsurgical varicocele repairs and vasectomy reversals precisely and artistically performed, ureteral and kidney stones removed without incisions. We are strongly connected to the quality of life of men who come to us, trying to help to urinate better, to improve sexual function and to become fathers. We are also patient when we wait for children with urological diseases to reach the appropriate age for surgery, often more than once. When we obey countless steps for the safe resection of cancer. And we aim to invade the patient as little as possible, applying robotic and laparoscopic techniques. Finally, if the cure is not possible, we know how to alleviate symptoms and we know how to support the patients when they need us the most. As urologists, we use cutting edge techniques combined with humanism, practicing Medicine at its best.

It was like urologists that we entered that ward. And that's how we were able to point out and support the patients who were on their way home and those who needed more care. That's how we called the rapid response team to timely assist intubation and to transfer the patients to the intensive care unit before a critical situation emerged. That's how we discussed the compassionate use of antivirals and several drugs. That's how we were able to refine treatments and standardize patient care. That's how we brought human values to our institution, providing not only improvement in the care of our ward but also the remainder of the hospital. That's how we could understand that all the dystopia we were experiencing could be small in the broader view of a lifetime. It was as urologists that with the colleagues of the internal medicine, day by day, we found the best therapeutic proposals for our patients. And as urologists we suffer when the first colleague of the team and then the first resident of urology was struck by COVID-19. Thus, we prayed for them to reunite with us the soonest, healthy, to keep assisting our patients. Until they don´t need us anymore.

Leaving our comfort zone reminded us that true resistance comes from resilience. We must not forget that even the best genetic characteristics are vulnerable to chance. How many will not have disappeared in a great storm, or under a meteor, before they are even selected? We don't know when and how it all will end, but our commitment is to what we can do now. We were trained to practice a patient-centered medical approach. Each individual we treat is a singular person and deserves the best management for the situation that he or she is facing at that moment. We are now entering a time in which we may need to change our patient-centered to a population-centered mentality. This kind of mindset shift is so disruptive that it must be avoided and postponed if possible not to collide with our principles as doctors and human beings. We will not allow chance to destroy our best characteristics. We urologists, human beings, can give transcendence to everything we are experiencing. And that is what we will continue to do.
